# Phenotypic features of *EYS*-associated retinitis pigmentosa with the c.2528 G > A (p.Gly843Glu) mutation in a Japanese cohort

**DOI:** 10.1038/s41598-026-46464-3

**Published:** 2026-04-03

**Authors:** Kensuke Muto, Ai Fujita Sajiki, Kensuke Goto, Yuki Kimura, Junya Ota, Hiroaki Ushida, Kazuhisa Yamada, Koji M. Nishiguchi, Taro Kominami

**Affiliations:** 1 Department of Ophthalmology, Nagoya University Graduate School of Medicine, 65 Tsurumai-Cho, Showa-Ku, Nagoya, Aichi 466-8560 Japan; 2 Medical Studies, School of Medicine, College of Health, Adelaide University, Adelaide, South Australia Australia; 3 Division for Advanced Medical Research, Center for Research of Laboratory Animals and Medical Research Engineering, Nagoya University Graduate School of Medicine, Nagoya, Aichi Japan

**Keywords:** *EYS*, Genotype, Retinitis pigmentosa, c.2528G > A, p.Gly843Glu, G843E, Diseases, Genetics, Medical research

## Abstract

Eyes shut homolog (*EYS)* is an eye-specific gene that encodes a protein essential for maintaining photoreceptor integrity. The aim of this study was to identify the parameters that characterize the clinical features of *EYS*-associated retinitis pigmentosa (RP) in patients carrying the c.2528G > A (p.Gly843Glu; G843E) variant. We retrospectively analyzed 127 Japanese patients carrying biallelic pathogenic variants in the *EYS* gene. To delineate the phenotypic impact of the hypomorphic G843E variant, the cohort was divided into the G843E group (*n* = 32) and the non-G843E group (*n* = 52). To account for age-related effects, clinical parameters—including best-corrected visual acuity (BCVA), Humphrey Field Analyzer mean deviation (MD), and ellipsoid zone (EZ) width—were compared between groups within the 40–60-year age range. The G843E group showed a significantly later age of disease onset (*p* = 0.0085), while BCVA and MD did not differ significantly between groups. Conversely, EZ width was significantly higher in the G843E group (*p* = 0.019), and multivariable logistic regression identified EZ width as the only statistically significant predictor of G843E variant presence (pseudo-R^2^ = 0.52). These results suggest that the G843E variant is associated with a milder clinical course, characterized by later onset and better preservation of photoreceptor structure. EZ width may serve as a key structural biomarker for genotype–phenotype correlations in *EYS*-RP.

**Supplementary Information:**

The online version contains supplementary material available at 10.1038/s41598-026-46464-3.

## Introduction

Retinitis pigmentosa (RP) is one of the most common inherited retinal dystrophies, affecting approximately one in 3000–5000 individuals worldwide^1^. RP is characterized by progressive retinal degeneration, with rod photoreceptor loss predominating in the early stages of the disease, leading to night blindness and peripheral vision impairment. As the disease progresses, cone photoreceptors also degenerate, leading to central vision loss. RP arises from highly heterogeneous genetic mutations, with over 80 different variants identified to date, encompassing autosomal dominant, autosomal recessive (arRP), and X-linked (XLRP) forms^2^. This genetic diversity contributes to substantial variability in age of onset, disease progression, and severity^3^.

Recent advances in comprehensive genetic analyses have facilitated rapid identification of genes associated with RP, revealing that the spectrum of gene variants differs across populations. Notably, variants in the eyes shut homolog (*EYS*) gene are among the most prevalent causes of arRP, accounting for 20%–40% or more of arRP cases in Japanese patients^4–8^. Therefore, the *EYS* gene represents a critical target for RP research in East Asian populations and for the development of new therapeutic approaches.

The *EYS* gene is located on chromosome 6q12, spanning a genomic region of over 2 Mb and comprising 44 exons. Its longest transcript isoform is 10,475 nucleotides in length and encodes a protein of 3165 amino acids, which includes a signal peptide, 28 epidermal growth factor-like domains, and 5 laminin G-like domains^9,10^. This protein represents the largest eye-specific structural protein identified to date, and, as its name suggests, is the human homolog of the Drosophila *EYS* protein^11^.

Studies using animal models have shown that *EYS* localizes to the connecting cilia of photoreceptor cells and plays an essential role in maintaining the integrity of photoreceptor outer segments^12,13^. Ablation of *EYS* in zebrafish leads to mislocalization of outer segment proteins and subsequent photoreceptor degeneration, confirming its essential role in preserving retinal structural stability and photoreceptor functioning^14^. In vertebrates, *EYS* is specifically expressed in photoreceptors. Immunolocalization analyses have confirmed the presence of *EYS* in the outer segments of porcine rod photoreceptors^10,15^. Additionally, studies using macaque retinal cryosections have confirmed *EYS* localization in the ciliary axoneme of both rod and cone photoreceptors, as well as in the cytoplasm of ganglion cells. These findings suggest that *EYS* contributes to maintaining ciliary axoneme stability within these anatomic structures^15^. In humans, pathogenic variants in the *EYS* gene also result in structural disruption of photoreceptor cells and have been identified as the pathogenic gene responsible for RP25^10^. Functional analyses in human retinal organoids have further clarified the pathogenic role of *EYS* in retinal dystrophy. In patient-derived organoids, *EYS* deficiency impairs outer segment protein localization and increases susceptibility to light-induced damage^16^.

RP exhibits substantial heterogeneity in both severity and clinical manifestations, depending on the mode of inheritance^3^. Even variants within the same gene can produce differences in disease progression and phenotype^6,17–20^. Truncating variants, such as nonsense and frameshift mutations, typically lead to loss of functional protein and are associated with earlier disease onset and more rapid progression^6,17^. Conversely, some missense mutations may retain residual function and are often associated with slower progression and later onset^21,22^. The missense variant G843E in the *EYS* gene is among the most commonly detected variants in Japanese patients with *EYS*-associated with RP (*EYS*-RP). In this population, *EYS* variants account for 46.6% of RP cases with phenotypic expression, with G843E representing 13.2% of these cases^4^. Previous studies have demonstrated a discrepancy between the relatively high allele frequency of G843E and its lower-than-expected observed prevalence in the RP population, suggesting that G843E does not cause complete loss of function but instead represents a hypomorphic allele^23,24^; however, no direct functional analyses or detailed clinical evaluations have quantified the extent to which outer retinal structure or visual function is preserved in patients with *EYS*-RP carrying pathogenic G843E variants.

Therefore, this study sought to clarify the differences in clinical manifestations between patients with and without the G843E variant in *EYS*-RP and to evaluate parameters relevant to genotype–phenotype correlations. By comparing age at onset, best-corrected visual acuity (BCVA), visual field sensitivity, and preservation of retinal structure between RP patients with and without the G843E variant, we quantitatively evaluated its phenotypic impact and improved the accuracy of genetic classification and prognosis assessment. Furthermore, given recent advances in genome editing and gene therapy, including CRISPR/Cas9 approaches^25^, understanding the functional impact of *EYS* variants is essential for patient stratification and the identification of appropriate therapeutic targets. This study may provide additional insights into the relationships between genotype and phenotype in *EYS*-RP and may contribute to future discussion on medicine in this condition.

## Results

### Patient characteristics

**Fig. 1:**
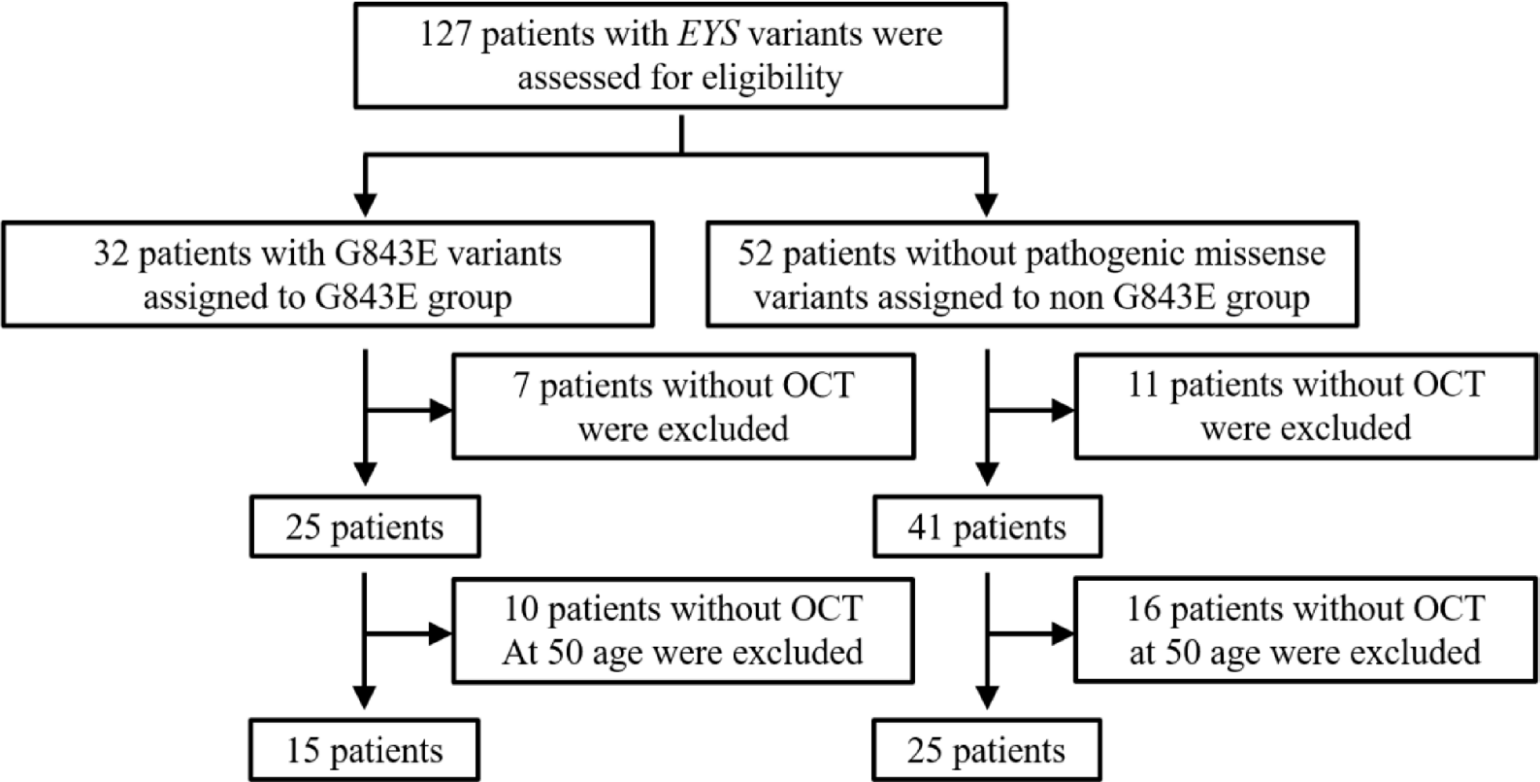
Flowchart of patient selection and assignment to *EYS* and non-*EYS* groups. A total of 127 patients with pathogenic *EYS* variants were enrolled in the analysis. Thirty-two patients with the c.2528G > A (p.Gly843Glu) variant were assigned to the G843E group, and 52 patients without pathogenic missense variants were assigned to the non-G843E group. In the G843E group, 7 patients without optical coherence tomography (OCT) data and an additional 10 without OCT data at approximately 50 years of age were excluded. In the non-G843E group, 11 patients without OCT data and 16 without OCT in the same age range were excluded. Consequently, 15 patients were assigned to the G843E group, and 25 were included in the non-G843E group.

The analysis included 127 patients with retinitis pigmentosa who harbored biallelic pathogenic or likely pathogenic *EYS* variants, as determined according to the Japanese interpretation of the ACMG guidelines^26^. Of these, 32 patients heterozygous for the c.2528 G > A (p.Gly843Glu) variant were classified into the G843E group, and 52 patients without pathogenic missense variants were assigned to the non-G843E group. The 52 patients without pathogenic missense variants carried other types of pathogenic variants, including truncating (nonsense or frameshift) and splice-site variants. Among the patients who underwent optical coherence tomography (OCT), the mean age was approximately 50 years; 15 were in the G843E group, and 25 were in the non-G843E group (Fig. 1). The *EYS* variants identified in the patients in both groups are listed in Table 1.

**Table 1:** EYS variants detected in patients of the G843E group and non-G843E group (NM_001142800.2).

Allele1	J-IRD-VI criteria/variant interpretation	Allele 2	J-IRD-VI criteria/variant interpretation	Number
G843E group				
c.2528 G > A (p.Gly843Glu)	PS3_Moderate, PM3_Very strong, PM5, PP3 / Pathogenic	c.4957dupA (p.Ser1653Lysfs*2)	PVS1, PS4, PM3_Very strong, PM1_Strong, PP5, BS1/Pathogenic	17
c.2528 G > A (p.Gly843Glu)	PS3_Moderate, PM3_Very strong, PM5, PP3/Pathogenic	c.8805 C > A (p.Tyr2935*)	PS4, PM3_Very strong, PP5, BS1/Pathogenic	4
c.2528 G > A (p.Gly843Glu)	PS3_Moderate, PM3_Very strong, PM5, PP3/Pathogenic	c.2826_2827delAT	PVS1, PS4, PM2, PM3, PP5/Pathogenic	1
c.2528 G > A (p.Gly843Glu)	PS3_Moderate, PM3_Very strong, PM5, PP3/Pathogenic	c.6563 T > C (p.Ile2188Thr)	PS4, PM3_Very strong, PM5_Supporting, PP5/Pathogenic	1
c.2528 G > A (p.Gly843Glu)	PS3_Moderate, PM3_Very strong, PM5, PP3/Pathogenic	c.7665_7666del (p.Tyr2555*)	PVS1, PS4, PM2, PM3/Pathogenic	1
c.2528 G > A (p.Gly843Glu)	PS3_Moderate, PM3_Very strong, PM5, PP3/Pathogenic	c.6714delT (p.Ile2239Serfs*17)	PVS1, PS4, PM3_Strong, PP5/Pathogenic	1
Non-G843E group				
c.4957dupA (p.Ser1653Lysfs*2)	PVS1, PS4, PM3_Very strong, PM1_Strong, PP5, BS1/Pathogenic	c.4957dupA (p.Ser1653Lysfs*2)	PVS1, PS4, PM3_Very strong, PM1_Strong, PP5, BS1/Pathogenic	10
c.4957dupA (p.Ser1653Lysfs*2)	PVS1, PS4, PM3_Very strong, PM1_Strong, PP5, BS1/Pathogenic	c.8805 C > A (p.Tyr2935*)	PS4, PM3_Very strong, PP5, BS1/Pathogenic	9
c.8805 C > A (p.Tyr2935*)	PS4, PM3_Very strong, PP5, BS1/Pathogenic	c.8805 C > A (p.Tyr2935*)	PS4, PM3_Very strong, PP5, BS1/Pathogenic	5
c.1211dupA (p.Asn404Lysfs*3)	PVS1, PS4, PM3_Strong, PP5 / Pathogenic	c.1211dupA (p.Asn404Lysfs*3)	PVS1, PS4, PM3_Strong, PP5/Pathogenic	2
c.4957dupA (p.Ser1653Lysfs*2)	PVS1, PS4, PM3_Very strong, PM1_Strong, PP5, BS1/Pathogenic	c.8805 C > A (p.Tyr2935*)	PS4, PM3_Very strong, PP5, BS1/Pathogenic	2
c.4957dupA (p.Ser1653Lysfs*2)	PVS1, PS4, PM3_Very strong, PM1_Strong, PP5, BS1/Pathogenic	c.6714delT (p.Ile2239Serfs*17)	PVS1, PS4, PM3_Strong, PP5/Pathogenic	2
c.4957dupA (p.Ser1653Lysfs*2)	PVS1, PS4, PM3_Very strong, PM1_Strong, PP5, BS1/Pathogenic	c.7665_7666del (p.Tyr2555*)	PVS1, PS4, PM2, PM3/Pathogenic	2
c.1211dupA (p.Asn404Lysfs*3)	PVS1, PS4, PM3_Strong, PP5 / Pathogenic	c.4957dupA (p.Ser1653Lysfs*2)	PVS1, PS4, PM3_Very strong, PM1_Strong, PP5, BS1/Pathogenic	1
c.410delA (p.Asn137Ilefs*5)	PVS1, PM2, PM3_Supporting/Pathogenic	c.4957dupA (p.Ser1653Lysfs*2)	PVS1, PS4, PM3_Very strong, PM1_Strong, PP5, BS1/Pathogenic	1
c.942delT (p.Ala315Leufs*24)	PVS1, PS4, PM3_Very strong/Pathogenic	c.6714delT (p.Ile2239Serfs*17)	PVS1, PS4, PM3_Strong, PP5/Pathogenic	1
c.1299 + 1 G > T (p.?)	PVS1, PM2, PM3, PP5/Pathogenic	c.4957dupA (p.Ser1653Lysfs*2)	PVS1, PS4, PM3_Very strong, PM1_Strong, PP5, BS1/Pathogenic	1
c.2023 + 1 G > T (p.?)	PVS1, PM2, PM3/Pathogenic	c.4957dupA (p.Ser1653Lysfs*2)	PVS1, PS4, PM3_Very strong, PM1_Strong, PP5, BS1/Pathogenic	1
c.2259 + 1 G > A (p.?)	PVS1, PS4, PM3_Strong, PM1_Strong, PP5/Pathogenic	c.4957dupA (p.Ser1653Lysfs*2)	PVS1, PS4, PM3_Very strong, PM1_Strong, PP5, BS1/Pathogenic	1
c.3243 + 1 G > A (p.?)	PVS1, PM2, PM3, PP5/Pathogenic	c.4957dupA (p.Ser1653Lysfs*2)	PVS1, PS4, PM3_Very strong, PM1_Strong, PP5, BS1/Pathogenic	1
c.4957dupA (p.Ser1653Lysfs*2)	PVS1, PS4, PM3_Very strong, PM1_Strong, PP5, BS1/Pathogenic	c.7919 C > A (p.Trp2640*)	PVS1, PS4, PM3_Very strong, PM1_Strong, PP5/Pathogenic	1
c.7665_7666del (p.Tyr2555*)	PVS1, PS4, PM2, PM3 Pathogenic	c.8805 C > A (p.Tyr2935*)	PS4, PM3_Very strong, PP5, BS1/Pathogenic	1

In the G843E group, there were no homozygous c.2528 G > A (p.Gly843Glu) patients, whereas homozygous c.4957dupA (p.Ser1653fs) patients were the most common in the non-G843E group. In silico prediction scores for missense and splice-site variants are summarized in Supplemental Table S1, and representative variants on the *EYS* domain structure are mapped (Supplemental Figure S1).

Table 2 presents the comparative clinical characteristics of patients in both groups. Sex distribution, family history, consanguinity, and initial symptoms did not differ significantly between groups. However, the age at symptom onset and the age at first clinical visit were significantly higher in the G843E group than in the non-G843E group (*p* = 0.0085 and *p* = 0.0017, respectively). Within the G843E group, the ages at first visit between the group carrying the c.4957dupA variant on the other allele and the group without the c.4957dupA were compared. The mean ages at first visit of these groups were 51.76 years and 44.63 years, respectively, and there was no significant difference between those two groups (*p* = 0.21, Mann–Whitney U test).

**Table 2:** Demographic information of patients.

Number of patients	G843E group	Non-G843E group	*P*-value
	25	41	
Median age at onset (IQR)	36.0 (26.5)	23.0 (18.0)	0.0085
Median age at first visit (IQR)	48.0 (21.0)	40.0 (17.0)	0.0017
Sex (male/female)	14 / 11	23 / 18	0.80
Family history +	5	11	0.74
Consanguineous marriage+	0	4	0.28
Symptom at onset			
Night blindness	12	30	0.072
Visual field loss	3	4	0.90
Others	10	7	0.076
BCVA at approximately 50 years of age in RE (logMAR(SD))	0.37 (0.48)	0.46 (0.65)	0.51
EZ width at approximately 50 years of age in RE (µm(SD))	2650 (2205)	1305 (891)	0.019
HFA MD value at approximately 50 years of age in RE (dB (SD))	−16.75 (9.86)	−19.86 (8.92)	0.44

IQR, interquartile range; P, probability; BCVA, best-corrected visual acuity; MAR, minimum angle of resolution; HFA, Humphrey field analyzer; MD, mean deviation; dB, decibel.

**Fig. 2:**
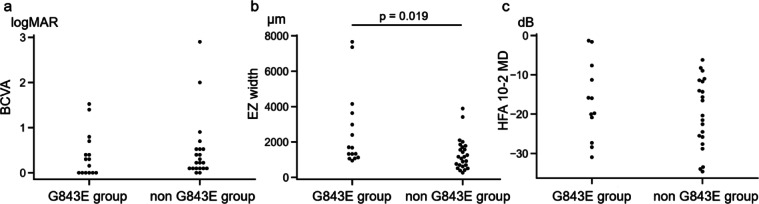
Comparison of visual parameters between the G843E and non-G843E groups. These panels show swarm plots of best-corrected visual acuity (BCVA) (logMAR) (**a**), ellipsoid zone (EZ) width (µm) (**b**), and the mean deviation value from the humphrey visual field 10–2 program (dB) (**c**) at approximately 50 years of age for the G843E and non-G843E groups. The difference in EZ width between the groups was statistically significant (Mann–Whitney U test, *p* = 0.019). MAR = minimum angle of resolution; dB = decibel.

Figure 2 shows the differences in clinical visual parameters between the two groups. BCVA at 50 years of age was 0.37 ± 0.48 (logMAR; mean ± standard deviation (SD)) in the G843E group and 0.46 ± 0.65 (logMAR; mean ± SD) in the non-G843E group (Fig. 2a). The mean deviation (MD) at 50 years of age was − 16.75 ± 9.86 (mean ± SD) in the G843E group and − 19.86 ± 8.92 (mean ± SD) in the non-G843E group (Fig. 2b). Although BCVA and MD values appeared better in the G843E group, these differences were not statistically significant. Conversely, the ellipsoid zone (EZ) width was significantly greater in the G843E group (2649.50 ± 2,204.57 μm; mean ± SD) than in the non-G843E group (1304.50 ± 890.66 μm; mean ± SD; *p* = 0.019) (Fig. 2c).

### Multiple regression analyses

The logistic regression model showed a good fit, with a pseudo-R^2^ of 0.52 and a log-likelihood of − 9.70. Among the predictor variables, the EZ width was the only statistically significant predictor of the presence of the G843E variant (*p* = 0.019; Table 3). The Akaike Information Criterion (AIC) value was 35.40, indicating good model fit. Variance inflation factor (VIF) values for all predictors were below 5, indicating a similar trend.

**Table 3:** Results of logistic regression with stepwise feature selection (Lasso).

Parameters	Coefficient	SE	z	*P*-value	95% confidence interval
BCVA at 50 years of age	2.35	1.41	1.67	0.095	[− 0.41, 5.11]
EZ at 50 years of age	4.03	1.81	2.23	0.026	[0.48, 7.58]
MD at 50 years of age	− 0.50	1.04	-0.48	0.63	[− 2.54, 1.55]
Male	3.39	2.03	1.67	0.095	[− 0.60, 7.38]
Visual field defects	− 5.13	4.22	−1.22	0.22	[− 13.41, 3.14]
Visual acuity loss	24.14	2.1 × 10^4^	0.0010	1.00	[− 4.11 × 10^4^, 4.12 × 10^4^]
No symptoms	2.96	1.64	1.80	0.072	[− 0.26, 6.18]

SE, standard error; BCVA, best-corrected visual acuity; EZ, ellipsoid zone; MD, mean deviation.

## Representative cases

**Fig. 3:**
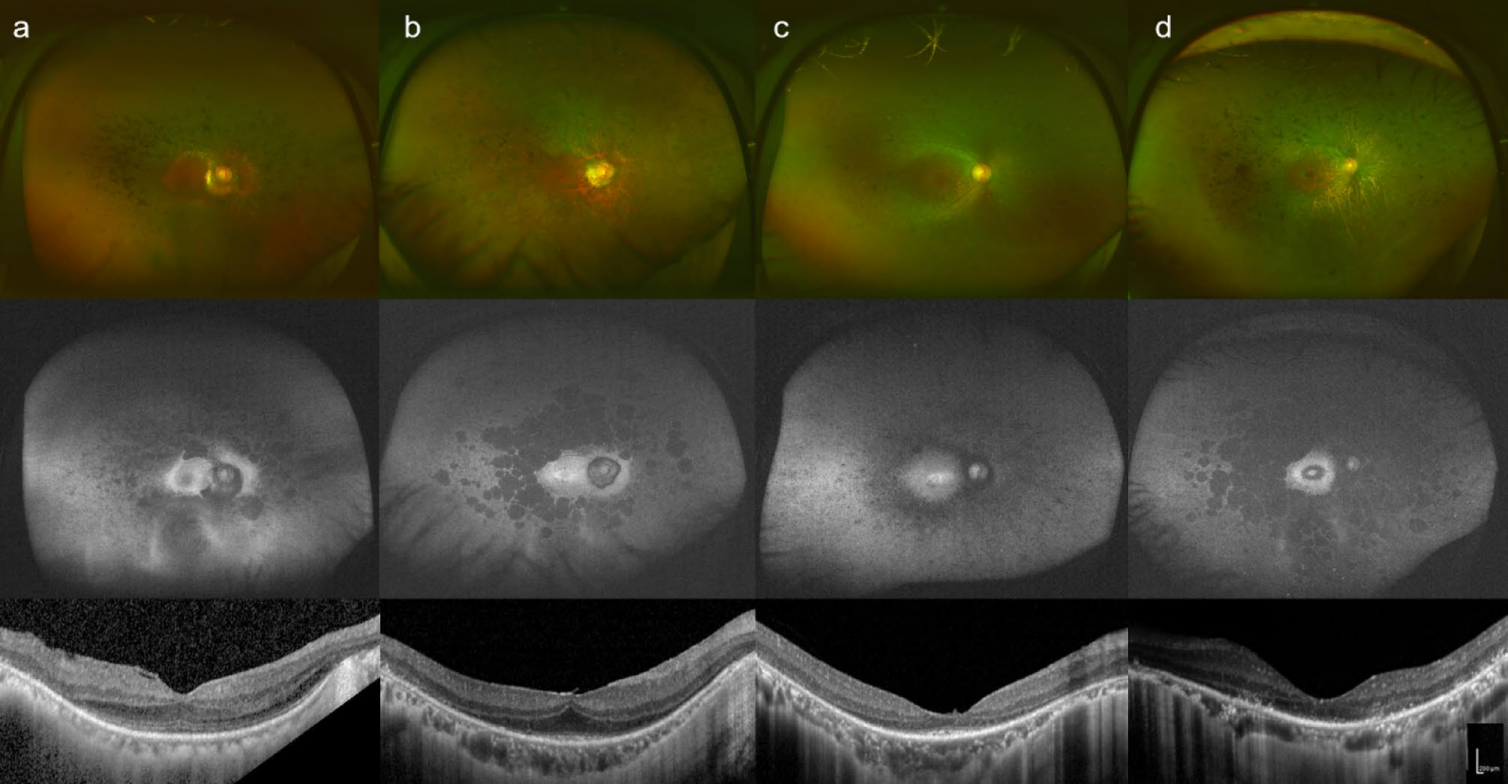
Fundus color photographs, fundus autofluorescence images, and horizontal OCT of representative cases. Each panel shows multimodal retinal imaging from the same patient, including fundus color photographs (top), fundus autofluorescence images (middle), and horizontal OCT images (bottom). Multimodal retinal imaging of (**a**) the right eye of a 54-year-old male patient (N1067) in the G843E group carrying compound heterozygous variants p.(Gly843Glu) and p.(Ser1653fs); (**b**) the right eye of a 50-year-old female patient (N427) in the G843E group with the same compound heterozygous variants as in panel (**a**); (**c**) the right eye of a 42-year-old female patient (N167) in the non-G843E group carrying a homozygous p.(Ser1653fs) variant; and (**d**) the right eye of a 51-year-old female patient (N559) in the non-G843E group carrying compound heterozygous variants p.(Tyr2555fs) and p.(Ser1653fs).

Wide-field fundus photographs, fundus autofluorescence (FAF) images, and horizontal OCT scans of representative cases are shown in Fig. 3. In both the G843E and non-G843E groups, fundus examinations revealed common features. Color fundus photographs showed retinal degenerative changes and vascular attenuation, while FAF images confirmed the presence of abnormal autofluorescence in all patients.

OCT scans also revealed shared characteristics, including outer retinal layer atrophy in both groups. However, a notable difference was observed in the EZ width near the fovea, which was consistently higher in the G843E group.

For instance, in the G843E group, the right eye of a 54-year-old male patient (N1067), carrying compound heterozygosity of p.(Gly843Glu) and p.(Ser1653fs), had an EZ width of 4164 μm (Fig. 3a). Another patient in this group, a 50-year-old female patient (N427) with the same compound heterozygous variants, had an EZ width of 1796 μm in her right eye (Fig. 3b).

Conversely, patients in the non-G843E group generally showed smaller EZ widths. The right eye of a 42-year-old female patient (N167) with a homozygous p.(Ser1653fs) variant had an EZ width of 1166 μm (Fig. 3c). Similarly, the right eye of a 51-year-old female patient (N559), carrying compound heterozygous variants p.(Tyr2555fs) and p.(Ser1653fs), had an EZ width of 1345 μm (Fig. 3d).

## Discussion

In this study, significant differences were observed in both age at onset and age at first clinical visit, with the G843E group exhibiting median differences of 13.0 and 8.0 years, respectively, compared with the non-G843E group (*p* = 0.0085 and 0.0017). Furthermore, after adjusting for age, the G843E group exhibited approximately twice the mean EZ width compared with the non-G843E group, a difference that was statistically significant (*p* = 0.019). These findings indicate that patients carrying G843E have a later disease onset and better preservation of the outer retinal structure, supporting the conclusion that this variant may be associated with milder clinical manifestations in *EYS*-RP.

The pathogenic mechanism of the G843E variant is hypothesized to involve conformational changes in the *EYS* protein resulting from its specific position and variant type. The G843E variant, a missense mutation located within an EGF-like domain, results in a glycine-to-glutamate substitution that may alter protein folding and extracellular interactions without causing complete loss of function. It is known that *EYS* is a central component of the complex responsible for maintaining the structural integrity of the outer retinal layers^10^.^12–15^ Previous reports suggest that the G843E variant represents a hypomorphic allele^23,24^ and may produce structural and functional phenotypes with relatively mild clinical manifestations, as compared to truncating variants, which are typically associated with near-complete loss of *EYS* protein function^6,18,22^. The EZ width data obtained in this study are consistent with these earlier reports, indicating the G843E variant likely preserves partial protein function, maintains photoreceptor structure, and manifests as a relatively slow-progressing disease. A plausible explanation for the milder structural phenotype observed in c.2528G > A carriers is that this missense allele retains partial *EYS* function. In an autosomal-recessive setting, a hypomorphic allele paired with a loss-of-function allele could still produce a small amount of functional protein, thereby slowing photoreceptor degeneration and preserving EZ width. The age at first visit did not significantly differ between patients carrying c.4597dupA on the other allele and those who did not, suggesting that the presence of one c.2528G > A allele may contribute to a relatively consistent clinical course across different trans-allele classes. However, the sample size limits our ability to detect modest trans-allele effects. Future studies integrating transcript-level assays and quantitative variant-effect prediction will be needed to define how much residual *EYS* activity is required to preserve photoreceptor structure. In larger cohorts with standardized OCT timepoints, these quantitative predictors could be examined in relation to EZ width to explore whether clinically useful thresholds can be defined.

In this study, the EZ width at approximately 50 years of age was significantly lower in the non-G843E group. On OCT, EZ width serves as a reliable marker of photoreceptor outer segment preservation and is commonly employed to quantify residual photoreceptors in retinal diseases with progressive course^27–30^. A reduction in EZ width is strongly associated with the progression of retinal degeneration^31,32^ and serves as a valuable indicator for assessing disease severity. Although one previous study reported a dissociation between structural and functional parameters in RP^33^, several studies have shown a strong correlation between EZ width and functional parameters, such as electroretinogram (ERG) responses and Goldmann visual field measurements^34^, confirming its potential as a structural biomarker. Moreover, the area of preserved EZ on OCT closely correlates with visual field sensitivity^35,36^, suggesting its potential utility for predicting visual field progression. Furthermore, subtle disease progression that may not be detectable by visual acuity or visual field tests can be captured through monitoring EZ disappearance on OCT imaging^37–39^. This underscores the high sensitivity of EZ assessment for detecting subtle changes in visual function that may not be apparent with conventional clinical evaluations, such as BCVA and Humphrey Field Analyzer (HFA) perimetry.

The results of the logistic regression analysis further support the potential of EZ width as a structural biomarker for identifying carriers of the G843E variant. In this analysis, multiple clinical parameters were evaluated as candidate predictors for G843E carrier status, and among them, only EZ width at approximately 50 years of age was identified as a statistically significant predictor (*p* = 0.019). The model showed a good fit, with a pseudo-R^2^ value of 0.52, a log-likelihood of − 9.70, and an AIC of 35.40. VIF values for all predictors were below 5, indicating negligible multicollinearity.

These findings suggest that EZ width may reflect genotype-associated differences with higher sensitivity than other clinical measures, such as visual acuity or visual field sensitivity, highlighting its relevance not only as an indicator of disease progression but also as a possible marker associated with specific genetic variants. Consistently, previous reports have shown that the rate of EZ width reduction differs significantly between ciliopathy-related and non-ciliopathy gene groups in arRP^40^. This suggests that EZ width serves as a structural parameter reflecting both disease progression and genotype-specific differences. These characteristics underscore its clinical utility for pathological classification and contribute to the foundation of personalized medicine approach.

One possible explanation for the lack of difference in visual acuity between groups, despite differences in EZ width, is that visual acuity primarily depends on macular integrity, whereas EZ width reflects photoreceptor preservation across a broader retinal region. Therefore, visual acuity may be reduced even when the EZ is relatively preserved if the fovea is compromised^41^. Visual acuity depends not only on the overall length of the EZ but also on the degree of fovea preservation. In patients with RP carrying the G843E variant, it can be inferred that even when the EZ is relatively well-defined, visual function may not correspondingly reflect this structural preservation.

Patients in whom photoreceptor structure is relatively preserved despite impaired visual function may represent promising candidates for gene therapy. The *EYS* gene exceeds the 4.7 kb packaging capacity of conventional adeno-associated virus (AAV) vectors^42,43^, rendering *EYS*-RP unsuitable for conventional AAV-mediated gene therapy. Alternatively, CRISPR–Cas9-based gene-editing technologies, such as base editing and prime editing, provide a feasible strategy for treating *EYS*-RP^44,45^.

Preservation of photoreceptor structure is essential for effective treatment^46^, and interventions at stages when the retinal structure remains intact are expected to yield more favorable outcomes. It is anticipated that some patients with RP carrying the G843E variant meet these structural criteria, representing a relevant population for timely therapeutic interventions.

In this study, patients carrying the G843E variant appeared to have slower disease progression than those lacking it. This finding suggests that the type of genetic mutation can influence the clinical course of *EYS*-RP and may inform future prognostic assessments and treatment selection based on mutational profiles^47^. Considering that this patient subgroup tends to exhibit delayed symptom onset and better preservation of photoreceptor structure, genetic screening can provide valuable prognostic information, supporting patient counseling, informed consent, and therapeutic decision-making. If personalized therapies, such as gene therapy or genome editing, become clinically available, the timing of intervention may differ depending on the genotype. Notably, some individuals carrying the G843E variant retained a relatively wide EZ and preserved visual acuity and visual field sensitivity even beyond 60 years of age. These observations suggest that the therapeutic time window may extend into later decades for some G843E carriers, whereas patients without G843E may require intervention at younger ages. Nevertheless, age alone is an imperfect surrogate, and structural preservation on OCT (e.g., EZ width) may represent a more practical criterion for defining treatable stages.

Another possible explanation for the absence of statistically significant differences in functional measures, such as visual acuity and MD values, is the limited sample size of the present study. These functional measures are subject to substantial variability and are highly influenced by individual differences, making it challenging to detect statistically significant differences in small cohorts. Therefore, the lack of statistically significant differences observed in this study does not preclude a potential trend toward relatively preserved visual function in patients carrying the G843E variant. Future large-scale, multicenter cohort studies with long-term follow-up will be necessary to clarify the impact of the G843E variant on visual function.

In this study, patients heterozygous for the G843E variant were compared to patients lacking pathogenic missense variants to evaluate its hypomorphic effect, excluding the possibility that other pathogenic missense variants may also retain variable residual function and produce heterogeneous phenotypes. Although c.8805 C > A (p.Tyr2935*) introduces a premature stop codon, it may not cause complete loss of function because it is located near the C terminus, leading to underestimation of the hypomorphic effect of the G843E variant. In the sub-analysis, patients heterozygous for the G843E variant were compared with patients carrying truncating variants other than c.8805 C > A (p.Tyr2935*), and the overall results were consistent with those of our previous analysis. Deletions and other structural variants in *EYS* have been reported. In this study, long-read sequencing was not performed, and there is a possibility of missing the effect of any deletion variants.

In conclusion, our study reveals that patients carrying the G843E variant in *EYS* exhibit milder clinical manifestations, including later symptom onset and relatively better preservation of photoreceptor structure, compared with patients lacking missense *EYS* variants. Notably, EZ width at approximately 50 years of age was significantly greater in patients with the G843E variant, supporting its possible utility as a sensitive structural biomarker that reflects genotype-associated differences in disease progression. These findings may highlight the importance of genetic characterization in *EYS*-RP, providing prognostic additional information that can inform patient counseling and guide individualized therapeutic planning. Furthermore, incorporating EZ width as a structural endpoint in clinical trials, especially for patients with early- to mid-stage disease, may enhance sensitivity for detecting treatment effects.

## Methods

We retrospectively analyzed the medical records of patients who attended the Ophthalmology Department at Nagoya University Hospital and were diagnosed with *EYS*-RP between March 2013 and March 2024. Written informed consent was obtained from all participants. The study protocol adhered to the Declaration of Helsinki and was approved by the Institutional Review Board and Ethics Committee of Nagoya University Hospital (study number 2020-0598). Data were accessed on February 16, 2025. Identifiable patient information was available during data collection; however, the dataset was anonymized prior to the conduct of this study.

### Clinical evaluations

RP was diagnosed by physicians specializing in hereditary retinal dystrophies, based on clinical symptoms including night blindness, photophobia, tunnel vision, and visual acuity loss, as well as fundus findings such as bone spicule pigmentation, vessel attenuation, and abnormal fundus autofluorescence. Degeneration of the outer retinal layers was assessed using OCT, and severely reduced responses on full-field ERGs were included in the diagnostic criteria. Enrolled patients were stratified into two groups: those heterozygous for the c.2528 G > A (p.Gly843Glu) variant (G843E group) and those without pathogenic missense variants (non-G843E group). Because other pathogenic missense variants may also retain variable residual function and produce heterogeneous phenotypes, patients heterozygous for the c.2528 G > A (p.Gly843Glu) variant were compared with patients lacking pathogenic missense variants to evaluate its hypomorphic effect. Differences between groups were evaluated for age at symptom onset and at first clinical visit, BCVA, EZ width, and HFA 10–2 MD. To validate the disease progression depending on the patients in the G843E group, the ages at first visit between the group carrying the c.4957dupA variant on the other allele and the group without the c.4957dupA were compared. To adjust for age-related effects, analyses of BCVA, EZ width, and MD were performed in patients aged closest to 50 years (between 40 and 60 years) and included only patients with OCT measurements within the range of age. OCT images were obtained using Spectralis OCT (Heidelberg Engineering, Heidelberg, Germany). EZ width was measured as previously described^30^, defined as a straight-line distance between the two points where the top of the EZ and the RPE intersect. An average of horizontal and vertical fovea-centered B-scan values was analyzed. Ultrawide-field color fundus photographs and fundus autofluorescence images were obtained using an Optos system (Optos P200Tx; Optos, Dunfermline, UK). Functional assessment was performed using standard automated perimetry with the Humphrey Field Analyzer (Carl Zeiss Meditec, Dublin, CA, USA).

### Genetic analyses

Genomic DNA was extracted from blood or saliva samples obtained from patients and analyzed using targeted resequencing of 86 genes with a next-generation sequencing platform, following methodologies described in previous reports^4,48^. The RefSeq transcript used for variant annotation was NM_001142800. Pathogenicity of detected variants was classified according to the J-IRD-VI guidelines^26^. Patients harboring *EYS* gene variants classified as pathogenic or likely pathogenic were included in the study. Cases with homozygous or compound heterozygous pathogenic variants were considered genetically resolved. For visualization, a schematic map of the *EYS* protein domains was generated, and the positions of representative variants detected in this cohort were annotated (Supplemental Figure S1). In silico prediction scores (REVEL, CADD PHRED, and SpliceAI DS_max) were obtained using the Ensembl Variant Effect Predictor and are presented in Supplemental Table S1. Protein-level consequences were described using HGVS nomenclature, including the predicted termination position for frameshift variants (fs*X).

### Statistical analysis

Differences in the proportions of patients with a family history, consanguinity, and symptoms at onset were assessed using the chi-square test. Continuous variables, including age at onset and at first clinical visit, BCVA, EZ width, and MD, were compared between groups using the Mann–Whitney U test. Multivariable logistic regression analysis was performed to examine the association between clinical parameters and the presence of the G843E variant. Predictor variables included BCVA at 50 years of age, EZ width at 50 years of age, gender, family history of disease, and symptom manifestations. These variables were selected using stepwise feature selection through Lasso regression with cross-validation. Before regression analysis, categorical variables were one-hot encoded, and continuous variables were standardized. VIF analysis was conducted to assess multicollinearity among predictors, and AIC was calculated to evaluate model fit. Statistical significance was defined as *p* < 0.05. Statistical analyses were conducted using Python SciPy and Statsmodels libraries^49^.

## Electronic Supplementary Material

Below is the link to the electronic supplementary material.

Supplementary Material 1

## Data Availability

The datasets generated and/or analyzed during the current study are available in the ClinVar repository. The specific variant reported in this study, *EYS* c.2528G> A (p.Gly843Glu), has been deposited under the accession number SCV004707555. These data, along with other related variants from this cohort (Submission ID: SUB14253519), can be accessed at (https://www.ncbi.nlm.nih.gov/clinvar/submitters/509444).
