# Supplementary material for: Phenotypic features of EYS-associated retinitis pigmentosa with the c.2528 G > A (p.Gly843Glu) mutation in a Japanese cohort
Source: Sci Rep. 2026 Apr 3;16:15906. doi: 10.1038/s41598-026-46464-3 (PMC13194756; doi:10.1038/s41598-026-46464-3)
Supplement: Supplementary file 1 — Supplementary Material 1 [file 41598_2026_46464_MOESM1_ESM.docx]

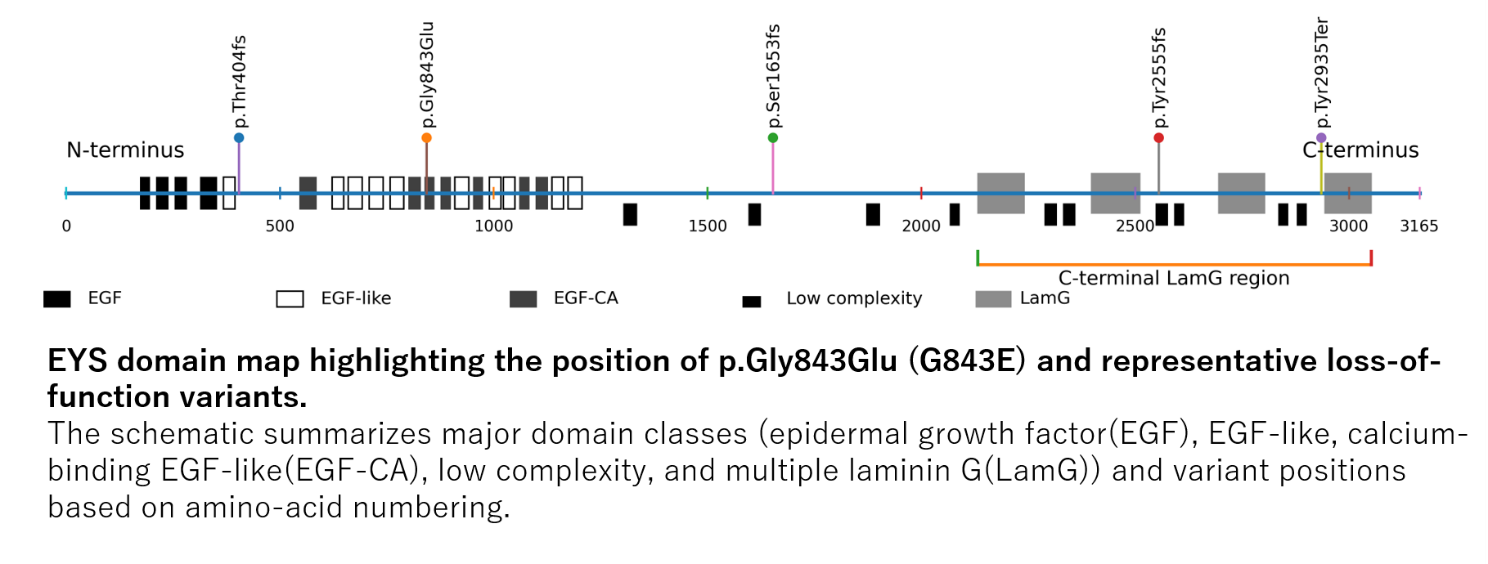
**Supplemental Figure S1. *EYS* domain map highlighting the position of p.Gly843Glu (G843E) and representative loss-of-function variants.**

The schematic summarizes major domain classes (epidermal growth factor (EGF), EGF-like, calcium-binding EGF-like(EGF-CA), low complexity, and multiple laminin G(LamG)) and variant positions based on amino-acid numbering.

Supplemental Table S1

| **In silico prediction scores for missense and canonical splice-site variants in *EYS*** | | | | | | | | |
| --- | --- | --- | --- | --- | --- | --- | --- | --- |
| Variants are described in HGVS notation based on RefSeq transcript NM_001142800.2. Allele counts correspond to the clinical-data subset analyzed in this study. | | | | | | | | |
|  |  |  |  |  |  |  |  |  |
| **cDNA (HGVS)** | **Protein (HGVS)** | **Variant class** | **Allele count (G843E group)** | **Allele count (non-G843E group)** | **Total alleles** | **REVEL** | **CADD PHRED** | **SpliceAI DS_max** |
| c.410delA | p.Asn137fs | Frameshift | 0 | 1 | 1 | NA | NA | NA |
| c.942delT | p.Ala315fs | Frameshift | 0 | 1 | 1 | NA | NA | NA |
| c.1211dupA | p.Asn404fs | Frameshift | 0 | 5 | 5 | NA | NA | NA |
| c.1299+1G>T | p.? | Splice donor | 0 | 1 | 1 | NA | NA | 1.0 |
| c.2023+1G>T | p.? | Splice donor | 0 | 1 | 1 | NA | NA | 0.92 |
| c.2259+1G>A | p.? | Splice donor | 0 | 1 | 1 | NA | NA | 0.98 |
| c.2528G>A | p.Gly843Glu | Missense | 25 | 0 | 25 | 0.73 | 23.1 | NA |
| c.2826_2827delAT | p.? | Indel (protein TBD) | 1 | 0 | 1 | NA | NA | NA |
| c.3243+1G>A | p.? | Splice donor | 0 | 1 | 1 | NA | NA | 0.99 |
| c.4957dupA | p.Ser1653fs | Frameshift | 17 | 42 | 59 | NA | NA | NA |
| c.6563T>C | p.Ile2188Thr | Missense | 1 | 0 | 1 | 0.352 | 19.99 | NA |
| c.6714delT | p.Ile2239fs | Frameshift | 1 | 3 | 4 | NA | NA | NA |
| c.7665_7666del | p.Tyr2555fs | Frameshift | 1 | 3 | 4 | NA | NA | NA |
| c.7919C>A | p.Trp2640* | Nonsense | 0 | 1 | 1 | NA | NA | NA |
| c.8805C>A | p.Tyr2935* | Nonsense | 4 | 22 | 26 | NA | NA | NA |
|  |  |  |  |  |  |  |  |  |
|  |  |  |  |  |  |  |  |  |
| Abbreviations: REVEL, Rare Exome Variant Ensemble Learner (0–1; higher values indicate higher predicted deleteriousness for missense variants); CADD, Combined Annotation Dependent Depletion (PHRED-scaled; larger values indicate higher predicted deleteriousness). SpliceAI DS_max is the maximum of four delta scores (acceptor gain/loss and donor gain/loss; 0–1). NA indicates not applicable or not interpreted for the given variant class (e.g., truncating variants). | | | | | | | | |
